# Skeletal Muscle Mitochondrial Bioenergetics and Morphology in High Fat Diet Induced Obesity and Insulin Resistance: Focus on Dietary Fat Source

**DOI:** 10.3389/fphys.2015.00426

**Published:** 2016-01-20

**Authors:** Rosalba Putti, Vincenzo Migliaccio, Raffaella Sica, Lillà Lionetti

**Affiliations:** Department of Biology, University of Naples “Federico II,”Naples, Italy

**Keywords:** mitochondrial fusion, mitochondrial fission, lard, fish oil, omega-3 fatty acids

## Abstract

It has been suggested that skeletal muscle mitochondria play a key role in high fat (HF) diet induced insulin resistance (IR). Two opposite views are debated on mechanisms by which mitochondrial function could be involved in skeletal muscle IR. In one theory, mitochondrial dysfunction is suggested to cause intramyocellular lipid accumulation leading to IR. In the second theory, excess fuel within mitochondria in the absence of increased energy demand stimulates mitochondrial oxidant production and emission, ultimately leading to the development of IR. Noteworthy, mitochondrial bioenergetics is strictly associated with the maintenance of normal mitochondrial morphology by maintaining the balance between the fusion and fission processes. A shift toward mitochondrial fission with reduction of fusion protein, mainly mitofusin 2, has been associated with reduced insulin sensitivity and inflammation in obesity and IR development. However, dietary fat source during chronic overfeeding differently affects mitochondrial morphology. Saturated fatty acids induce skeletal muscle IR and inflammation associated with fission phenotype, whereas ω-3 polyunsaturated fatty acids improve skeletal muscle insulin sensitivity and inflammation, associated with a shift toward mitochondrial fusion phenotype. The present minireview focuses on mitochondrial bioenergetics and morphology in skeletal muscle IR, with particular attention to the effect of different dietary fat sources on skeletal muscle mitochondria morphology and fusion/fission balance.

## Introduction

Skeletal muscle seems to play a central role in whole body insulin resistance (IR) and metabolic syndrome associated with high fat (HF) feeding, obesity and aging (see Corpeleijn et al., 2009; DeFronzo and Tripathy, 2009; Lark et al., 2012).

**Figure 1:**
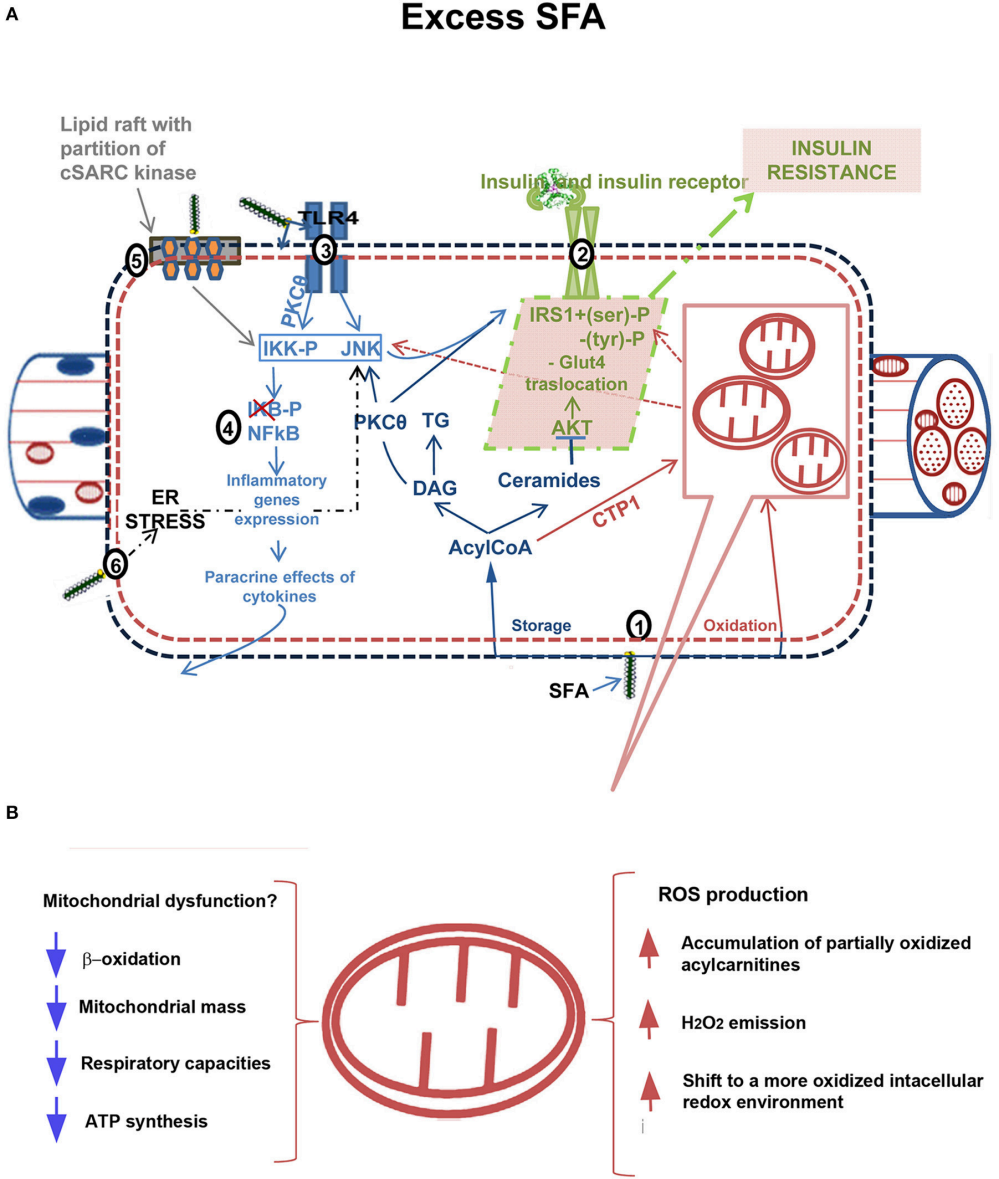
**Mechanism linking excess fatty acids to insulin resistance in skeletal muscle. (A):** (1) Excess free fatty acids (FFAs) are esterified in AcylCoAs, substrates involved in both synthetic and oxidative pathways. In the synthetic pathway, they are either stored as Triacylglycerols (TG) in lipid droplets or accumulated in metabolites (DAGs, Ceramides) that act as signaling molecules and may interfere with normal cellular signaling. DAGs are associated with membrane translocation and activation of Protein kinase C theta (PKC-θ), increased IRS1 serine/threonine phosphorylation and decreased insulin-stimulated IRS1 tyrosine phosphorylation, whereas Ceramides impair insulin action by inhibiting protein kinase PKB/AKT (dark blue). In the oxidative pathway, AcylCoAs are imported into the mitochondria by carnitine palmitoyltransferase-1 (CPT-1) shuttle and degraded via β-oxidation (in purple). (2) Insulin signaling pathway impaired by excess FFA (in green). Among FFAs, saturated FAs (SFAs) stimulate the activation of several inflammatory pathways. (3) Receptor-mediated mechanisms, as those of Toll like receptors 2/4 (TLR 2/4), activate serine kinases inhibitor kappaB kinase (IKK) and c-JUN NH_2_-terminal kinase (JNK). The activation of PKCθ also contributes to IKK and JNK activation. Altogether, these kinases catalyze IRS1 serine phosphorylation and lead to a reduction of insulin-induced IRS1 tyrosine phosphorylation, impairing insulin action. Moreover, IKK/NFkB axis (4) triggers expression of inflammatory genes with cytokines production (e.g., Tumor necrosis factor, TNFα), which in turn activate intracellular pathways promoting insulin resistance development (in light blue). (5) SFAs enter the cellular membranes and incorporate into them reducing membrane fluidity and creating or expanding subdomains rich in cholesterol and sphingolipids (lipid raft). They induce clustering and activation of cytosolic cSRC. cSRC activity is required for JNK1 activation and inhibition of insulin signaling (in grey). (6) Endoplasmic reticulum stress (ER stress), induced by lipotoxicity, contributes to activate inflammatory pathways and impair insulin signaling. **(B)**: Putative role of mitochondria in development of IR. Mitochondrial dysfunction in presence of excess FFAs leads to intramyocellular lipid accumulation due to impaired β-oxidation. Decreased mitochondrial mass, respiratory capacities and ATP synthesis have been found in obesity and diabetes. Alternatively, excess FFA within mitochondria in the absence of increased energy demand stimulates oxidative stress with high rates of ROS production and H_2_O_2_ emission and a shift to a more oxidized intracellular redox environment, ultimately leading to the development of IR.

Some evidence suggested that cytosolic ectopic accumulation of fatty acids (FA) metabolites, such as diacylglycerols (DAG) and/or Ceramides, (Yu et al., 2002; Adams et al., 2004), underlies IR development in skeletal muscle (lipotoxicity theory) (reviewed in Lark et al., 2012). Numerous evidence has also suggested a link between elevated systemic and tissue inflammation with IR (inflammatory theory) (see Shenk et al., 2008; Lark et al., 2012). The effectors of IR in HF diet-induced inflammation are suggested to involve hyperactivation of stress-sensitive Ser/Thr kinases, such as JNK and IKKβ, which in turn inhibits insulin receptor/IRS1 axis. Several mechanisms were proposed to explain the link between inflammation and IR: endoplasmic reticulum (ER) stress (Ozcan et al., 2004; Lionetti et al., 2009; Mollica et al., 2011), oxidative stress (Lark et al., 2012), signaling through inflammation-associated receptors, such as TLR4 signaling (Uysal et al., 1997; Shi et al., 2006), and partitioning/activation of c-SRC (a key mediator of JNK activation) by saturated FA (Holzer et al., 2011; Figure 1A).

In recent years, different reviews focused on mechanism(s) by which mitochondrial bioenergetics (Fisher-Wellman and Neufer, 2012; Lark et al., 2012; Muoio and Neufer, 2012; Holloszy, 2013) and morphology (Liesa and Shirihai, 2013; Montgomery and Turner, 2015) may be linked to the etiology of IR in skeletal muscle. In the present review, the challenging debate on the involvement of mitochondrial dysfunction in IR will be briefly reviewed. Then, the main aim of the review will be to underline the importance of including mitochondrial morphology/dynamics and dietary fat source in the debate on skeletal muscle mitochondria involvement in IR etiology and to highlight the need of further research studies to clarify the involved mechanism(s).

## Skeletal muscle mitochondrial bioenergetics and IR

Two leading theories on mechanisms underlying skeletal muscle IR onset focus on mitochondria, although with opposite views (Figure 1B). In one theory, mitochondrial dysfunction is suggested to cause intramyocellular lipid accumulation leading to IR (Kelley et al., 1999; Lowell and Shulman, 2005; reviewed in Civitarese and Ravussin, 2008; Montgomery and Turner, 2015). In this case, the strategies to accelerate flux through β-oxidation should improve insulin sensitivity. In the second theory, excess fuel within mitochondria in the absence of increased energy demand stimulates mitochondrial oxidant production and emission, ultimately leading to the development of IR (Fisher-Wellman and Neufer, 2012; Figure 1B). In this case, elevated flux through β-oxidation without added energy demand is viewed as an underlying cause of IR disease (Muoio and Neufer, 2012).

Several studies have revealed a reduction in skeletal mitochondrial mass in obesity and type 2 diabetes (Kelley et al., 2002; Morino et al., 2005; Ritov et al., 2005), decreased ATP synthesis in insulin resistant offspring of patients with type 2 diabetes (Petersen et al., 2004, 2005) and decreased maximal respiration rates in skeletal muscle isolated mitochondria from type 2 diabetics (Mogensen et al., 2007). Moreover, with the limitation that gene expression is not a direct assessment of function itself, HF diet has been shown to coordinately down-regulate genes required for mitochondrial oxidative phosphorylation in human and rodents skeletal muscle (Sparks et al., 2005). Interestingly, in skeletal muscle from obese or diabetic patients, decreased activity of electron transport chain and reduced number of mitochondria have been mainly reported in skeletal muscle mitochondria located beneath the sarcolemmal membrane (SS mitochondria) (Ritov et al., 2005). SS mitochondria also displayed lower respiratory capacities in presence of succinate as substrates in adult rats exhibiting HF diet-induced IR (Lionetti et al., 2007). The two mitochondrial subpopulations (SS and intermyiofibrillar, IMF) are differentiated not only by the different localization but also by the different functions (Cogswell et al., 1993; Mollica et al., 2006): SS mitochondria could be more affected by the impairing effect of saturated FA due to their localization beneath the sarcolemmal membrane.

However, although correlative studies seem to implicate mitochondrial dysfunction and impaired β-oxidation as predisposing risk factors for IR, still uncertain is whether diminished fat oxidation reflects a cause or a late stage consequence of the disease process (reviewed in Muoio, 2010). In fact, obese/diabetic humans never use their mitochondrial capacity for lipid oxidation; therefore, a marginal decline in oxidative potential has little relevance in causing lipotoxicity and IR in sedentary individuals. Moreover, it has been suggested that early stages of obesity and IR are accompanied by increased, rather than reduced β-oxidation (Muoio, 2010). These findings question the concept that mitochondrial dysfunction is a primary cause of IR (Hoeks et al., 2010, 2011), as also underscored by the study of Bonnard et al. (2008), showing mitochondrial dysfunction in skeletal muscle after 16 weeks, but not after 4 weeks HF feeding, while muscle IR was observed after both 4 and 16 weeks of HF feeding. In addition, mitochondrial deficiency, severe enough to impair fat oxidation in resting muscle, cause an increase, not a decrease, in insulin action (reviewed in Holloszy, 2013). Altogether, these studies suggest that deficiency of mitochondria in muscle does not cause IR (reviewed in Holloszy, 2013).

An alternative mechanism to explain the connection between mitochondria and IR focused on reactive oxygen species (ROS) production (reviewed in Muoio and Neufer, 2012; Holloszy, 2013). Lefort et al. (2010) showed normal oxidative capacity, decreased mitochondrial mass and high rates of ROS production in mitochondria isolated from skeletal muscle of obese insulin resistant individuals. Moreover, FA overload within the mitochondria results in the accumulation of partially oxidized acyl-carnitines, increased mitochondrial hydrogen peroxide (H_2_O_2_) emission and a shift to a more oxidized intracellular redox environment (Anderson et al., 2009; reviewed in Lark et al., 2012). H_2_O_2_ emission may induce IR by directly targeting protein involved in the glucose uptake process. On the other hand, given the sensitivity of cellular phosphatase to redox state, it has been suggested that elevated mitochondrial H_2_O_2_ production may decrease phosphatase tone in cells, increasing the inhibition state of insulin signaling proteins by stress-sensitive kinases (reviewed in Lark et al., 2012).

Although the primary role of skeletal muscle mitochondrial dysfunction in the pathogenesis of IR and type 2 diabetes is under debate (Hoeks et al., 2010, 2011; Holloszy, 2013), it is generally accepted that in this disease a mitochondrial defect occurs, possibly secondary to a fat intake increase.

## Mitochondrial morphology and skeletal muscle IR

It is well known that mitochondrial morphology is highly variable, ranging between long tubular mitochondria and short circular ones and it is maintained through a dynamic balance between fusion and fission processes (Westermann, 2010, 2012), which allow mitochondria to redistribute in a cell, exchange contents and repair damaged mitochondria. These two opposing processes are finely regulated by mitochondrial fusion proteins mitofusins 1 and 2 (Mfn1 and Mfn2), and optic atrophy gene 1 (OPA1) (Cipolat et al., 2004; Palmer et al., 2011) and by mitochondrial fission protein dynamin-related protein 1 (DRP1) and fission protein 1 (Fis1) (Nunnari et al., 2002; Liesa et al., 2009).

Several pieces of evidence suggested that mitochondrial dynamic behavior play a key role in mitochondrial health, bioenergetics function, quality control, and cell viability. Notably, disruption of mitochondrial dynamics has been found in IR and type 2 diabetes (Bach et al., 2003, 2005; Yu et al., 2006; Liesa and Shirihai, 2013).

The group of Zorzano showed that decreased expression of Mfn2 and altered expression of OPA1 participated in obesity and type 2 diabetes development in both patients and rodent models (Bach et al., 2003; Zorzano et al., 2009a,b, 2010; Hernández-Alvarez et al., 2010; Quirós et al., 2012). In obese Zucker rats, skeletal muscle mitochondrial network was reduced by 25% associated with a repression of Mfn2 (Bach et al., 2003). In addition, skeletal muscle of obese subjects and type 2 diabetic patients also showed a reduced expression of Mfn2 mRNA and Mfn2 protein compared to lean subjects (Bach et al., 2003, 2005). Mfn2 repression was detected in the skeletal muscles of both obese and non-obese type 2 diabetic patients (Bach et al., 2005). Notably, the expression of one of the mitochondrial proteases involved in OPA1 processing, presenilin-associated rhomboid-like (PARL), was also reduced in diabetic animals. In humans, a positive linear correlation between PARL mRNA levels and insulin sensitivity has been reported (Walder et al., 2005). These data suggest multiple alterations in mitochondrial fusion in IR. However, reduction of Mfn2 expression together with decreased mitochondrial size in skeletal muscle in obesity and type 2 diabetes states allow proposing a relevant role for Mfn2 in IR (Civitarese and Ravussin, 2008; Zorzano et al., 2009a,b). In agreement with this suggestion, a positive correlation between Mfn2 expression in skeletal muscle and insulin sensitivity has been reported (Bach et al., 2005). It is of interest that the involvement of Mfns in diet-induced obesity via the regulation of leptin resistance and systemic energy metabolism was also revealed (Dietrich et al., 2013; Schneeberger et al., 2013; reviewed in Putti et al., 2015). Moreover, it has been suggested that there is an association between increased mitochondrial fission, mitochondrial bioenergetics and fat induced-IR in skeletal muscle (Jheng et al., 2012). Indeed, in differentiated C2C12 muscle cells mitochondrial fragmentation and increased mitochondrion associated-DRP1 and Fis1 was induced by excess palmitate and this fission phenotype was associated with increased oxidative stress, loss of ATP production and reduced insulin-stimulated glucose uptake. These authors also found smaller and shorter mitochondria and increased mitochondrial fission machinery in the skeletal muscle of mice with genetic or diet-induced obesity. Furthermore, inhibition of mitochondrial fission improved muscle insulin signaling and systemic insulin sensitivity in obese mice (Jheng et al., 2012).

A shift toward fission was also found in skeletal muscle of HF diet (HFD)-induced obese mice by Liu et al. (2014). Notably, these authors also faced the question of whether mitochondrial dynamics exists in skeletal muscle *in vivo*. It should be considered that mitochondria in skeletal muscle are rigidly located between bundles of myofilaments in a highly regular “crystal like” pattern (Vendelin et al., 2005) and therefore, their motility and dynamics may be very restricted. Liu et al. (2014) confirmed that mitochondria are dynamic organelles in skeletal muscle *in vivo*, by demonstrating that they exchange contents within the whole mitochondrial population through nanotunneling-mediated mitochondrial fusion. In this way, mitochondria can bypass the restriction of myofilament and exchange mitochondrial matrix contents even if they are distant. This dynamic communication among mitochondria in skeletal muscle may protect from injury by preventing the accumulation of detrimental metabolites. Liu et al. (2014) also showed that this dynamic behavior was inhibited in skeletal muscle of HFD–induced obese mice associated with decreased Mfn2 and increased Fis1 and DRP1 expression compared to normal diet fed mice. This impaired mitochondrial fusion in skeletal muscle of HFD-induced obese mice was accompanied with damaged mitochondrial respiratory function and decreased ATP content. Therefore, the authors suggest that mitochondrial dynamics play an important role in regulating mitochondrial function, including respiration rate and ATP production (Liu et al., 2014).

## Dietary fat source differently affect skeletal muscle IR and mitochondrial morphology

It has been suggested that diverse dietary fat sources have different effects on obesity and associated diseases development. Saturated FA are well known to induce both obesity and related disease, whereas omega 3 polyunsaturated FA (ω-3 PUFA) from fish oil have been shown in many studies to protect against these metabolic diseases (Xin et al., 2008; Gonzalez-Periz et al., 2009; Abete et al., 2011). The effect of ω-3 PUFA on metabolic disease has been extensively studied during the past three decades since the first studies such as the one by Storlien et al. (1987) showing that fish oil prevents IR induced by high-fat feeding in rats. Further studies confirmed that ω-3 PUFA had an anti-obesity effect and enhanced insulin sensitivity and glucose homeostasis in rodent models of IR: the replacement of a small proportion of the diet with ω-3 PUFAs from fish oil completely prevents the development of skeletal muscle IR (Storlien et al., 1991; Fryer et al., 1995). Recent studies hypothesized that ω-3 PUFAs protect glucose tolerance, in part by preventing the accumulation of bioactive lipid mediators that interfere with the insulin signaling pathway (Lanza et al., 2013). Lanza et al. (2013) evaluated the influence of dietary ω-3 PUFAs on mitochondrial physiology and muscle lipid metabolites in the context of 10 weeks high-fat feeding in mice. They found a lower content of long-chain Acyl-CoAs and Ceramides in the presence of fish oil, whereas mitochondrial oxidative capacity was similarly increased with or without fish oil. Several studies have also indicated that ω-3 PUFAs possess anti-inflammatory properties that prevent and reverse the development of IR in mice which are fed a high-fat diet in an adiponectin-dependent manner (Kalupahana et al., 2010, 2011). On the other hand, unsaturated FA prevent c-Src membrane partitioning and activation and block JNK activation with consequent beneficial effects on insulin sensitivity (Holzer et al., 2011). Considering the anti-inflammatory properties of ω-3 PUFAs, in a recent study, we compared the effects of chronic high-fish oil and high-lard diets on obesity-related inflammation by evaluating serum and tissue adipokine levels and histological features in insulin-sensitive tissues (white adipose tissue, liver and skeletal muscle) (Lionetti et al., 2014b). We showed that the replacement of lard (saturated FA) with fish oil (ω-3 PUFAs) in chronic high-fat feeding attenuated the development of systemic and tissue inflammation. Indeed, compared with a high-lard diet, a high-fish oil diet resulted in a lower degree of systemic inflammation and IR that were associated with a lower ectopic lipid depot, inflammation degree and IR in the skeletal muscle (Lionetti et al., 2014b). In a further study on the same experimental design, we confirmed that the replacement of lard with fish oil in HF diet had preventive effects on obesity and systemic inflammation and IR development as well as we showed a fusion mitochondrial phenotype in association with the improvement of IR in skeletal muscle (Lionetti et al., 2013). As for preventive effects on obesity, body weight gain after 6 weeks of HF diet was lower in fish oil fed rats compared to lard fed rats. As for skeletal muscle IR, we showed that high-lard diet induced a defect in the skeletal muscle insulin signaling pathway with a lower immune-reactivity to IRS1 and pIRS (Tyr632), in agreement with other authors (Yaspelkis et al., 2009; Yuzefovych et al., 2013). On the other hand, a high fish oil diet elicited IRS1 and pIRS (Tyr632) immune-reactivity similar to a control diet, in agreement with ameliorated systemic insulin sensitivity (Lionetti et al., 2013). We cannot exclude the possibility that the fish oil protective effect was due to indirect effects of differences on adiposity. We also showed that the beneficial effects of fish oil feeding on skeletal muscle IR development was associated with changes in protein involved in mitochondrial dynamic behavior, with a greater number of immunoreactive fibers for Mfn2 and OPA1 proteins, as well as a weaker immunostaining for DRP1 and Fis1 compared to high lard feeding. Skeletal muscle electron microscopy observations also suggested a prominent presence of fission events in high-lard diet fed rats, and fusion events in high-fish oil diet fed rats (Lionetti et al., 2013).

The finding on the effects of different dietary FA on skeletal muscle mitochondrial fusion/fission proteins may be associated with effects on inflammatory processes involved in IR development. Indeed, Bach et al. (2005) suggested that TNF-α inhibits Mfn2 gene expression in cells in culture, suggesting that inflammatory parameters may play a regulatory role on Mfn2. In agreement, we showed that pro-inflammatory dietary saturated FA reduced Mfn2 expression and induced fission phenotype in skeletal muscle (Lionetti et al., 2013). On the other hand, the anti-inflammatory effect of dietary ω-3 PUFAs was associated with no reduction in skeletal muscle Mfn2 content and a tendency to mitochondrial fusion. This shift toward fusion may be an adaptive mechanism to counteract cellular stress induced by chronic HF diet, by allowing functional mitochondria to complement dysfunctional mitochondria. In accordance, myocytes cultured with docosahexaenoic acid exhibited a higher mitochondrial mass with a higher proportion of large and elongated mitochondria with downregulation of fission genes DRP1 and Fis1 (Casanova et al., 2014).

The pro-fusion effect of ω-3 PUFAs dietary fat on skeletal muscle mitochondria is in agreement with the results found in liver mitochondria, where a shift toward mitochondrial fusion phenotype was also suggested (Zhang et al., 2011; Lionetti et al., 2014a).

The mechanism underlying fish oil/ω-3 PUFAs mitochondrial fusion stimulation may involve receptor-mediated signaling and/or lipid membrane composition, among other factors. Indeed, ω-3 PUFAs are incorporated into cellular membranes and may affect lipid-protein interactions as well as membrane fluidity, and therefore the function of embedded proteins. Recently, it has been suggested a role for ω-3 PUFAs in reorganizing the composition of the mitochondrial membrane, while promoting improvements in ADP sensitivity, determined as mitochondrial responses during ADP titration (Herbst et al., 2014). Moreover, it is well known that saturated FA incorporation reduces membrane fluidity, whereas PUFA do not have such effect (Clamp et al., 1997; Stulnig et al., 2001; Holzer et al., 2011). Further studies are needed to elucidate these mechanisms.

## Conclusions

**Figure 2:**
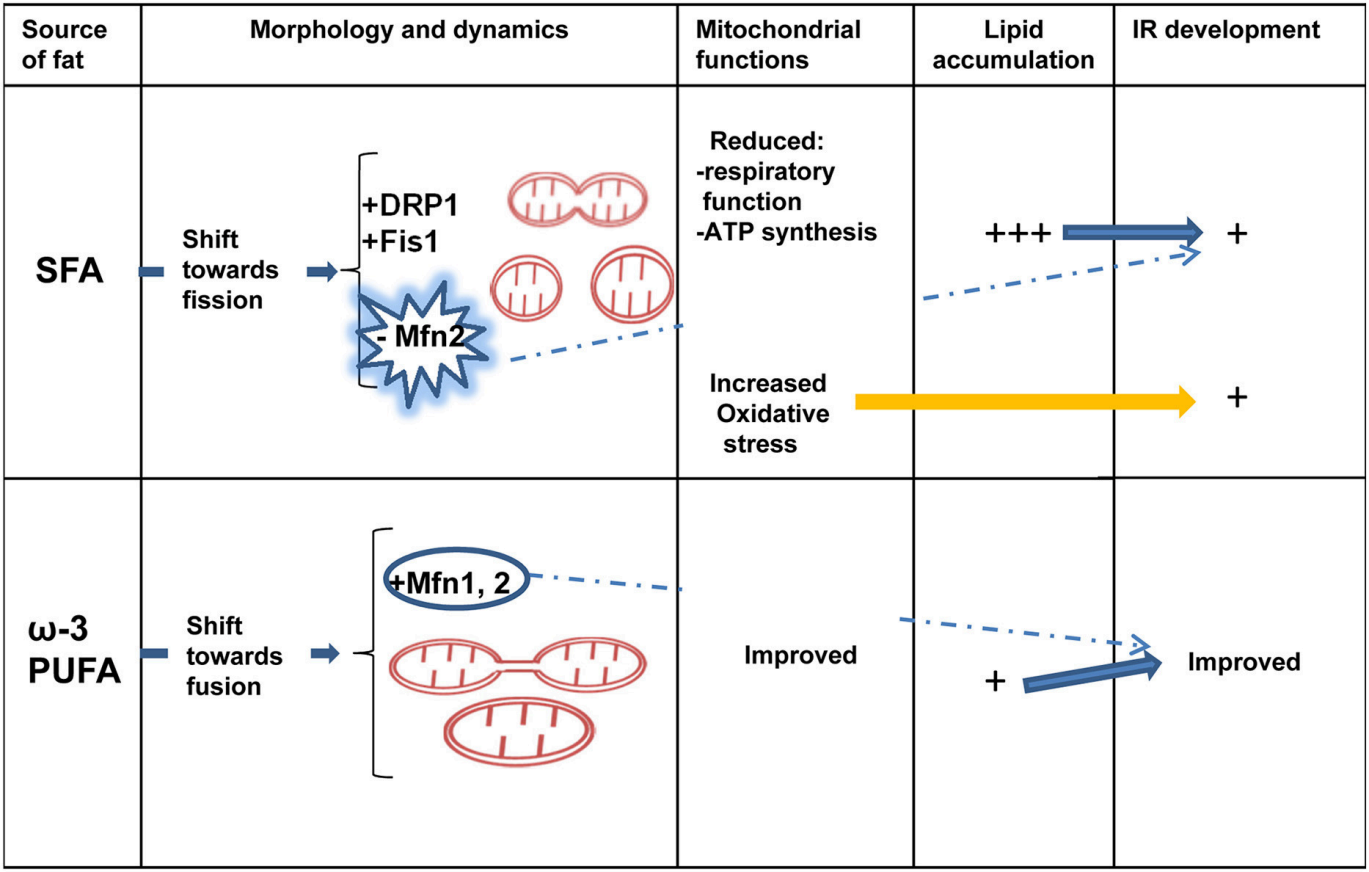
**Dietary fat source, mitochondrial dynamics and skeletal muscle IR**. Diverse dietary fat sources induce different effects on mitochondrial morphology/dynamics and IR development in skeletal muscle. Saturated fatty acids (SFA) elicit a shift toward fission processes (decreased Mfn2 and increased Fis1 and DRP1 expression) associated with reduced mitochondrial function, increased oxidative stress, lipid accumulation and IR development. Ω-3 polyunsaturated fatty acids (PUFAs) induce Mfn2 expression and fusion processes associated with less lipid accumulation and improved IR. IR, insulin resistance; Mfn2, mitofusin 2; DRP1, dynamin-related protein 1; Fis1, fission protein 1.

Recent evidence highlighted an association between mitochondrial morphology and IR development in skeletal muscle. Few works on different dietary fat source started to underline the different effect of saturated and ω-3 PUFAs on skeletal muscle IR and mitochondrial protein involved in dynamics behavior, suggesting an association between beneficial protective effect of ω-3 PUFAs toward IR development and mitochondrial fusion phenotype (Figure 2). However, it is important to underline that most of the data present in literature on skeletal muscle mitochondrial morphology and IR are from associational studies. Therefore, there is an urgent requirement for *in vivo* mechanistic studies to confirm the associational relationship between mitochondrial morphology/dynamics and IR development.

### Conflict of interest statement

The authors declare that the research was conducted in the absence of any commercial or financial relationships that could be construed as a potential conflict of interest.
